# Validation and Analysis of the Psychometric Properties of Two Irritability-Measuring Tools: The Affective Reactivity Index (ARI) and the Born-Steiner Irritability Scale (BSIS) in the Italian Adult and Adolescent Populations

**DOI:** 10.3390/ijerph20054607

**Published:** 2023-03-05

**Authors:** Maria Letizia Grassi, Donatella Valente, Anna Berardi, Marco Tofani, Giovanni Galeoto

**Affiliations:** 1 Department Human Anatomy, Histology, Forensic Medicine and Orthopedics, Sapienza University of Rome, 00185 Rome, Italy; 2 Department of Human Neurosciences, Sapienza University of Rome, 00185 Rome, Italy; 3 Neuromed IRCCS, 86007 Pozzilli, Italy

**Keywords:** irritability, adolescents, adulthood, psychometric properties, validity, reliability

## Abstract

Irritability is a transdiagnostic symptom that affects quality of life during the lifespan of individuals. The objective of the present research was to validate two assessment tools, namely the Affective Reactivity Index (ARI) and the Born-Steiner Irritability Scale (BSIS). We investigated internal consistency as measured with Cronbach’s alpha, test–retest with intraclass correlation coefficient (ICC) and convergent validity confronting ARI and BSIS scores with the strength and difficulties questionnaire (SDQ). Our results revealed ARI good internal consistency with a Cronbach’s α of 0.79 for adolescent and 0.78 for adults. The BSIS also demonstrated good internal consistency for both samples with Cronbach’s α = 0.87. Test–retest analysis showed excellent values for both tools. Convergent validity showed positive and significant correlation with SDW, albeit weak for some sub-scales. In conclusion, we found ARI and BSIS to be good tools for measuring irritability in adolescents and adults, and now, Italian healthcare professionals can use it with more confidence.

## 1. Introduction

Irritability is defined as a low threshold for experiences of anger in response to frustration, often associated with verbal and/or physical aggression [1]. During a recent seminar on childhood irritability, experts identified two components of irritability: tonic and phasic [2,3]. The former was defined as persistently angry, short-tempered, or grumpy mood, whereas the latter is defined as waves of intense anger, and behavioral outbursts in response to frustration [4]. The term “irritability” more frequently refers to the irritable mood rather than to tonic irritability, whereas terms such as “reactive aggression” and “impulsive aggression” were used to describe the intense manifestations of anger in phasic irritability. These two dimensions of irritability were defined as distinct constructs in terms of prediction of long-term clinical outcomes, response to treatment, or family history. Being able to draw a distinction between these two components of chronic irritability and untangle their heterogeneity is fundamental to clarifying the subtypes of the disorder and to conducting future investigations on the response to treatment [5].

Longitudinal and cross-sectional studies showed that chronic irritability, which includes both tonic and phasic components, is associated with decreased income, education, and an increased risk of anxiety, depression, and suicidal ideation [5]. It is important to highlight that tonic and phasic irritability are the major components of a disruptive mood dysregulation disorder (DMDD), recently introduced in the Diagnostic and Statistical Manual of Mental Disorders, 5th edition (DSM-5) [4,5]. These two components make up the two main symptoms of DMDD: severe, recurrent outbursts of anger (i.e., phasic irritability) and outbursts of irritability or angry mood (i.e., tonic irritability) [2].

Irritable children and adolescents show an orientation towards threatening environmental stimuli and tend to interpret ambiguous stimuli as threatening, with an increased risk of outbursts of anger and aggressive behavior (reactive aggression) [4]. The frequency of tantrums is very high during preschool and follows a well-defined trajectory; after preschool, irritability levels tend to be relatively stable over time, and this is similar to documented levels for anxiety and depression [1].

The DSM-5 mentions irritability in numerous disorders with greater prevalence in childhood; for instance, DMDD, Oppositional Defiant Disorder; furthermore, irritability is also a symptom of multiple disorders in adulthood (generalized anxiety disorder, bipolar disorder, cyclothymic disorder, major depression, and post-traumatic stress disorder [3]). In the DSM-5, the absence of a specific diagnosis of irritability for adults stands out; in fact, those manifesting symptoms of irritability in adulthood often receive alternative and incorrect diagnoses that do not exactly correspond to their specific clinical condition. This leads patients to feeling misunderstood and mislabeled; consequently, they do not experience authentic representations of their lived experiences [3]. Furthermore, adult irritability is often comorbid with other disorders with which it shares genetic and environmental antecedents and risk factors.

Irritability was inconsistently measured in the scientific literature, mainly due to the lack of clarity in the conceptualization of this construct [5]. Recently, the development of irritability scales significantly increased, demonstrating the renewed scientific interest in this field. However, there are several limitations to these current measurement tools, the first one concerning the definition of the concept of irritability. Second, despite specific outcome measures being available in Italy, there is still a need for specific validated instruments, such as the clinician-affective reactivity index (CL-ARI) [6] and the irritability in adult patients with epilepsy (I-Epi) [7] However, the CL-ARI is a clinician-rated instrument to assess irritability in pediatric research and clinical settings, while the I-Epi is an outcome measure specific for the condition of epilepsy. Two measures were found to be valid and reliable for general population: the affective reactivity index (ARI) and the Born-Steiner irritability scale (BSIS) [8]. Considering the need of specific assessment tools for general population is urgently required for both adults and adolescents, the primary objective of this study was to validate and analyze the psychometric properties of both ARI and BSIS in the Italian context. 

## 2. Materials and Methods

This study was conducted following the the consensus-based standards for the selection of health measurement instruments (COSMIN) [9] by a research group from Sapienza University of Rome and the Association of “Rehabilitation and Outcome measure Assessment” R.O.M.A. [10,11,12,13,14,15]. While the Italian version of the ARI was available, the research group—upon receiving consent from the developers—produced a translation and cross-cultural adaptation of the BSIS following the Rome Foundation guidelines.

### 2.1. Population

Samples were composed by a group of adolescents and adults. For sample adequacy, different studies recommended an item-to-response ratio varying from 1:3 to 1:20. Considering the BSIS had 14-items, and after analyzing the sample size of other validation studies (133–218), we considered recruiting a minimum of 280 people to be adequate. Adolescents were recruited from several Italian secondary schools. Parents/guardians agreed to give their consent. At the same time, for the adult population, we recruited some parents of the adolescent sample, as well as a convenience sample from general practitioners and university population. To participate, people had to meet the following inclusion criteria: a good understanding of the Italian language, no cognitive disabilities, no diagnosed psychiatric disorders or drug use. The inclusion criteria were assessed during specific meetings organized within the school or other facilities, where research purposes, timing, and administering approaches were explained. Those who showed interest in participating in the study and met the inclusion criteria were interviewed using ARI, BSIS, and SDQ. Data were collected using a web-app for storing data. Consequently, after a period ranging from 24–48 h, participants received a link to perform the re-test. 

### 2.2. Assessment Tools

The affective reactivity index (ARI) [16] is a concise tool developed to measure irritability in childhood and adolescence. The scale is available in two versions: one for self-assessment and the other for parents. ARI investigates three dimensions of irritability: threshold for an irritability reaction, frequency of irritability feelings/behaviors, and duration of such behaviors/feelings. Each item had three possible answers: “Not true”, “Quite true”, “Certainly true”, respectively, evaluated as 0, 1, 2. Only the first six items were added together to obtain the total score, while the seventh element, referring to the impairment determined by irritability, was examined separately. Total ARI scores by parent and self-report showed a Cronbach’s alpha of 0.92 and 0.88 in USA and 0.89 and 0.90 in UK, for the parent- and self-report scales, respectively [16]. For the present investigation, we used the Italian version available from King’s College London (https://www.kcl.ac.uk/academic-psychiatry/about/departments/child-adolescent-psychiatry, accessed on 28 February 2023).

The Born-Steiner irritability scale (BSIS) [17] was created for the assessment of irritability in the female population with mood disorders related to the menstrual cycle, pregnancy, or menopause. The BSIS was subsequently used to assess irritability also in the general population and other health conditions. An observer reported rating scale and a self-report rating scale were developed. For the present investigation, we used the 14-item self-report scale; scoring was attributed using a 4-point Likert scale. BSIS demonstrated good internal consistency (Cronbach’s α = 0.92) and acceptable test–retest reliability (r = 0.70).

The strength and difficulties questionnaire (SDQ) [18] is a brief emotional and behavioral questionnaire for the clinical assessment of children and young people. The tool can capture the perspective of children and young people, their parents, and teachers. There are currently three versions of the SDQ: a short form, a longer form with an impact supplement (which assesses the impact of difficulties on the child’s life), and a follow-up form. The 25 items in the SDQ comprise 5 scales of 5 items each. The scales include: (1) emotional symptoms subscale (2) conduct problems subscale (3) hyperactivity/inattention subscale (4) peer relationships problem subscale (5) prosocial behavior subscale. The SDQ can be completed by children and young people aged 11–17 years old, and a separate version can be completed by those aged 18 and over. SDQ is widely used in different countries and revealed good psychometric properties [19]. For the present investigation, we used the 25-item SDQ Italian version [20].

### 2.3. Psychometric Properties

All statistical analyses were performed with Statistical Package for Social Science (SPSS) 27.0. First, for both ARI and BSIS, we performed an exploratory factor analysis (EFA) with maximum likelihood factoring. The appropriateness of sampling was evaluated using the Keiser–Meyer–Olkin (KMO) and Bartlett’s test of sphericity. Subsequently, the reliability and validity for the BSIS and ARI scales were assessed considering the consensus-based standards for the selection of health measurement tools (COSMIN) [9]. The internal consistency was examined with Cronbach’s α to evaluate the interrelation of the items and the homogeneity of the scale. A value of α ≥ 0.7 is commonly considered to be acceptable [21]. To evaluate the test–retest reliability, a randomized sample of the whole population performed a second administration, two days apart [22]. This timing was considered adequate for measuring irritability without facing any clinical change. The intraclass correlation coefficient (ICC) was calculated to determine the degree to which repeated measurements were free from measurement errors. ICC values equal to or greater than 0.70 is commonly considered acceptable [21]. To assess convergent validity, the Italian version of the skills and difficulties questionnaire (SDQ-Ita) was administered to the entire population. The correlation between both ARI and BSIS scores and those of SDQ-ita was evaluated with Pearson’s correlation coefficients; a value greater than 0.5 indicates an acceptable level of correlation. where positive value means positive linear correlation, and a negative value means negative linear correlation [21,23]. *p* values < 0.05 were considered statistically significant. At the end, a Student *t*-test was used to explore differences in ARI and BSIS scores across gender.

## 3. Results

### 3.1. Translation and Cultural Adaptation of the BSIS

Once the consent of BSIS authors was received, the scale, originally in English, was translated into Italian, following international guidelines [24]. In order to adapt the word-for-word translated version to the Italian culture, a focus group reviewed the final translation, corrected any remaining incoherencies, and reformulated some elements to minimize differences from the original English version.

### 3.2. Participants

For the validation of the BSIS scale and the ARI scales, a sample of 435 adolescents and 174 adults was recruited, whose demographic characteristics are summarized in Table 1.

### 3.3. Factor Analysis 

ARI, KMO, and Bartlett’s test of sampling adequacy revealed a value of 0.847 with *p* < 0.01. The factor analysis extracted one factor that explained 57.12% of variance. BSIS, KMO, and Bartlett’s test of sampling adequacy revealed a value of 0.869 with *p* < 0.01. The factor analysis extracted one factor that explained 40.07% of variance.

### 3.4. Reliability and Validity of the BSIS and the ARI

The statistical analysis was carried out on 435 adolescents and 174 adults participating in the study, who answered to ARI, BSIS, and SDQ-ita in the same session. For test–retest, we sent a web link for answering to each assessment tool; however, the number of people participating in this second phase decreased from 435 to 224 for the adolescent group, and from 174 to 59 for adult population. BSIS demonstrated good internal consistency for both samples with Cronbach’s α = 0.79, (*p* < 0.05) for adolescents and Cronbach’s α = 0.78, (*p* < 0.05) for adults. Internal consistency was calculated for the full scale for both samples; the item-total correlation showed positive results as reported in Table 2.

The ARI also demonstrated good internal consistency for both samples with Cronbach’s α = 0.87, (*p* < 0.05) for adolescents and Cronbach’s α = 0.87, (*p* < 0.05) for adults. Internal consistency was calculated for the full scale for both samples; item-total correlation showed positive results as reported in Table 3.

Statistical analysis showed that both scales were comparable to the original version for both samples. For convergent validity, the Italian version of the SDQ questionnaire was distributed to the entire population. Pearson’s correlation coefficient showed statistically significant values for a positive correlation between the ARI scale and the SDQ-Ita subscales in both the adolescent and adult samples, thus indicating good convergent validity. Pearson’s correlation coefficient also showed statistically significant values for a positive correlation between the BSIS scale and the SDQ-Ita questionnaire in both the adolescent and adult samples, indicating good convergent validity. Pearson’s correlation coefficients are shown in Table 4.

The test–retest reliability, for both scales, was evaluated on the group of participants for whom it was possible to record data at t1 (224 adolescents and 59 adults). ARI obtained a CCI of 0.81 (*p* < 0.05) for adolescents and 0.93, (*p* < 0.05) for adults, respectively. BSIS achieved a CI of 0.90 (*p* < 0.05) for adolescents and 0.95, (*p* < 0.05) for adults. Both BSIS and ARI, therefore, showed good test–retest reliability, as shown in Table 5.

Finally, we also investigated specific differences between both BSIS and ARI across gender. Table 6 and Table 7 report mean (SD) values for each item of subscales with significant differences.

## 4. Discussion

Irritability recently became a widely discussed topic in the scientific literature due to the heterogeneity of manifestations and for its relationship with mental health and specific clinical conditions [24]. In addition, irritability can be exacerbated in specific environmental conditions. For instance, a recent study stressed the importance of focusing on irritability exhibited by children and adolescents with a diagnosis of autism spectrum disorder during COVID-19 home confinement [25]. However, despite this growing interest, knowledge on how to measure and treat irritability did not reach a universal and well recognized standard [26].

The present study aimed to contribute towards a better understanding of irritability. Our findings suggested that the Italian version of both BSIS and ARI are reliable and valid tools for assessing irritability in healthy Italian adult and adolescent population. Internal consistency of the ARI revealed a Cronbach’s α = 0.87 for both adolescents and adult population, in line with the Australian [27], the Brazilian [28], and the original [16] versions. For BSIS, we found a Cronbach’s α = 0.79 for adolescents and Cronbach’s α = 0.78 for the adult population, slightly lower than the original version [17]. However, our findings revealed BSIS and ARI as consistent tools to measure irritability in the target population. Furthermore, both tools also revealed good test–retest reliability: ARI obtained an ICC of 0.81 and 0.93 for adolescents and adult population, respectively. Our findings were in line with the Australian validation of the ARI [27], while other versions did not report test–retest reliability. BSIS obtained an ICC of 0.90 for adolescents and 0.95 for adult population, slightly higher than the original version [17]. Our findings proved that both ARI and BSIS are reliable measure confirming their stability over the time. Pearson’s correlations indicated that there were moderate to strong relationships between ARI and BSIS total score and SDQ-ita. Our analysis also proved moderate correlations between ARI scores and SDQ-ita subscales except for prosocial behavior, as reported in Australian validation study [27].

Regarding differences in ARI and BSIS scores among gender, we found specific characteristics in both adolescent and adult samples. We explored the effect of gender on irritability and found that women reported greater irritability when measured with both ARI and BSIS. Our findings were in line with previous studies of higher irritability rates among females rather than males [8]. The same differences in irritability rates were found in various population, such as in adolescents from general population [29], adult people with epilepsy [7], students with depressive symptoms [30], and adults with major depressive disorder [31,32]. These differences were more evident in adolescent populations; in fact, small but significant gender differences in emotion expressions were reported for adults [33], with women showing greater emotional expressivity, especially for positive emotions and internalizing negative emotions such as sadness. Gender differences in irritability and fear in response to a social stress challenge were consistent with previous studies that demonstrated that women were more likely to endorse negative effects than men in response to interpersonal stressors [34].

Finally, it is important highlight that irritability is one of the most transdiagnostic constructs in the Diagnostic and Statistical Manual of Mental Disorders, 5th edition (DSM-5), ranging across 15 disorders including mood disorders; trauma- and stressor-related disorders; disruptive, impulse-control, and conduct disorders; substance-related and addictive disorders; and personality disorders (American Psychiatric Association, 2013). However, an overlap in the conceptualizations of irritability, anger, and aggression exists and the failure to distinguish these symptoms presents several problems. First, it leads to the contamination of measures of irritability with items that represent other constructs. Second, it reduces the validity of research on these three constructs [5]. Therefore, our study, with the validation of two measurement tools, aimed to contribute to a better definition for measuring irritability in both adults and adolescents. However, this study had some limitations. First of all, the relatively small sample size to investigate test–retest reliability. Second, we did not conduct a power analysis for sample size and this can limit the generalizability of results. Future studies with larger samples should be conducted to examine how irritability can predict, for example, the clinical outcome in specific health conditions. 

## 5. Conclusions

The BSIS and the ARI were proven to be valid, reliable, easy-to-use tools for measuring irritability. In particular, the BSIS evaluated episodes of irritability in the last six months, and the ARI in the last week. These reasons make both tools suitable for epidemiological studies. The flexibility and specificity of the scales identified in this study allow the use of these tools in a wide range of clinical and research for both adults and adolescents.

## Figures and Tables

**Table 1:** Demographic characteristics of the adolescent and adult population recruited for the validation of the ARI and BSIS scales.

	**Adolescents**	**Adults**
No.	435	174
Mean age (SD)	12.22 (0.96)	41.37 (15.69)
Min–Max age	10–14	19–69
**Gender (%)**		
Males	212 (48.7)	30 (18.4)
Females	223 (51.3)	142 (81.6)

**Table 2:** Alpha deleted analysis of the BSIS scale in the adolescent and adult population.

	Adolescents					Adults				
	Medium Scale after Item Exclusion	Scale Variance after Item Exclusion	Item-Total Correlation	Quadratic Multiple Correlation	Alpha after Item Exclusion	Medium Scale after Item Exclusion	Scale Variance after Item Exclusion	Item-Total Correlation	Quadratic Multiple Correlation	Alpha after Item Exclusion
ITEM1	8.07	41.910	0.648	0.457	0.859	7.17	27.759	0.501	0.389	0.872
ITEM2	8.30	40.160	0.647	0.477	0.858	7.64	25.624	0.683	0.569	0.863
ITEM3	8.40	42.936	0.492	0.323	0.866	7.74	27.516	0.447	0.379	0.875
ITEM4	8.66	43.266	0.487	0.337	0.867	7.98	28.385	0.394	0.257	0.876
ITEM5	8.40	40.304	0.703	0.548	0.855	7.69	25.361	0.785	0.654	0.858
ITEM6	8.49	41.172	0.634	0.453	0.859	7.84	26.765	0.582	0.482	0.868
ITEM7	7.97	43.851	0.296	0.205	0.879	6.94	26.277	0.467	0.314	0.876
ITEM8	8.64	44.549	0.358	0.238	0.873	7.62	26.729	0.518	0.324	0.871
ITEM9	8.77	44.196	0.459	0.322	0.868	7.79	27.196	0.421	0.317	0.877
ITEM10	8.37	40.257	0.671	0.520	0.857	7.56	25.850	0.586	0.422	0.868
ITEM11	8.69	42.743	0.592	0.451	0.862	8.03	27.905	0.493	0.410	0.873
ITEM12	8.15	41.988	0.526	0.366	0.865	7.12	25.476	0.556	0.429	0.870
ITEM13	8.67	44.470	0.392	0.259	0.871	7.91	26.840	0.594	0.524	0.868
ITEM14	8.43	40.914	0.617	0.453	0.860	7.68	25.295	0.671	0.594	0.863

**Table 3:** Alpha deleted analysis of the ARI scale in the adolescent and adult population.

	Adolescents					Adults				
	**Medium Scale after Item Exclusion**	**Scale Variance after Item Exclusion**	Item-Total Correlation	Quadratic Multiple Correlation	Alpha after Item Exclusion	Medium Scale after Item Exclusion	Scale Variance after Item Exclusion	Item-Total Correlation	Quadratic Multiple Correlation	Alpha after Item Exclusion
ITEM1	2.63	6.380	0.502	0.271	0.766	1.31	3.361	0.493	0.271	0.763
ITEM2	2.31	5.638	0.634	0.423	0.739	1.29	3.143	0.597	0.504	0.739
ITEM3	2.60	6.401	0.438	0.241	0.779	1.40	3.550	0.412	0.211	0.780
ITEM4	2.85	6.869	0.521	0.304	0.768	1.62	4.283	0.375	0.208	0.785
ITEM5	2.52	5.941	0.591	0.391	0.749	1.41	3.437	0.584	0.386	0.743
ITEM6	2.55	5.911	0.584	0.357	0.750	1.49	3.526	0.596	0.455	0.742
ITEM7	2.71	6.747	0.383	0.194	0.787	1.49	3.503	0.604	0.444	0.740

**Table 4:** Pearson’s correlation coefficients between the Italian version of the ARI and BSIS scales and the SDQ-Ita questionnaire.

	ARI		BSIS	
	Adolescents	Adults	Adolescents	Adults
SDQ. Emotional problems	0.489 **	0.457 **	0.526 **	0.634 **
SDQ. Behavioral problems	0.605 **	0.408 **	0.535 **	0.328 **
SDQ. Problems with peers	0.555 **	0.480 **	0.507 **	0.499 **
SDQ. Hyperactivity	0.184 **	0.410 **	0.264 **	0.430 **
SDQ. Prosocial problems	−0.184 **	−0.171 *	−0.143 **	−0.233 *
SDQ. wqTotal	0.654 **	0.643 **	0.655 **	0.723 **

* *p* < 0.05; ** *p* < 0.01.

**Table 5:** Intraclass correlation coefficients for test–retest reliability of the ARI and BSIS scales in the adolescent and adult population.

	Adolescents					Adults				
	Mean (SD) Test	Mean (SD) Retest	ICC	CI [95%]	*p*	Mean (SD) Test	Mean (SD) Retest	ICC	CI [95%]	*p*
ARI	2.74 (2.52)	2.75 (2.59)	0.81	0.75–0.86	<0.05	1.11 (1.46)	0.95 (1.51)	0.93	0.88–0.96	<0.05
BSIS	9.16 (7.09)	8.46 (6.54)	0.89	0.86–0.91	<0.05	7.22 (5.17)	7.04 (5.84)	0.95	0.91–0.97	<0.05

**Table 6:** Gender differences in BSIS score.

		Adolescents			Adults		
BSIS	Gender	Mean	SD	*p*	Mean	SD	*p*
ITEM1	Male	0.84	0.669	<0.01 **	0.90	0.876	0.84
	Female	1.24	0.803		0.96	0.424	
ITEM2	Male	0.52	0.789	<0.01 **	0.30	0.483	0.25
	Female	1.02	0.975		0.51	0.626	
ITEM3	Male	0.54	0.748	<0.05 *	0.20	0.422	0.08
	Female	0.77	0.769		0.49	0.549	
ITEM4	Male	0.28	0.622	<0.01 **	0.00	0.000	>0.05
	Female	0.61	0.849		0.16	0.367	
ITEM5	Male	0.44	0.704	<0.01 **	0.30	0.483	0.35
	Female	0.89	0.920		0.47	0.588	
ITEM6	Male	0.35	0.675	<0.01 **	0.20	0.422	1
	Female	0.73	0.863		0.20	0.405	
ITEM7	Male	0.86	0.942	<0.01 **	0.80	0.919	0.07
	Female	1.35	0.926		1.29	0.727	
ITEM8	Male	0.39	0.727	0.48	0.50	0.527	0.95
	Female	0.46	0.773		0.49	0.589	
ITEM9	Male	0.23	0.571	<0.05 *	0.10	0.316	0.02 *
	Female	0.43	0.802		0.44	0.693	
ITEM10	Male	0.51	0.828	<0.001 **	0.40	0.516	0.35
	Female	0.88	0.923		0.62	0.716	
ITEM11	Male	0.30	0.646	0.50	0.00	0.000	0.02 *
	Female	0.35	0.602		0.20	0.588	
ITEM12	Male	0.81	0.821	<0.05 *	0.70	0.823	0.13
	Female	1.09	0.909		1.11	0.745	
ITEM13	Male	0.43	0.732	0.13	0.10	0.316	0.38
	Female	0.30	0.599		0.29	0.661	
ITEM14	Male	0.46	0.706	<0.01 **	0.20	0.422	0.05
	Female	0.84	0.957		0.56	0.755	
TOT	Male	6.94	6.644	<0.01 **	4.70	3.831	<0.05 ^*^
	Female	10.94	6.958		7.78	5.295	

* *p* < 0.05; ** *p* < 0.01.

**Table 7:** Gender differences in ARI score.

		Adolescents			Adults		
ARI	Gender	Mean	SD	*p*	Mean	SD	*p*
ITEM1	Male	0.28	0.471	<0.01 **	0.20	0.422	0.56
	Female	0.49	0.646		0.31	0.557	
ITEM2	Male	0.59	0.655	<0.01 **	0.30	0.483	0.95
	Female	0.84	0.685		0.31	0.514	
ITEM3	Male	0.34	0.573	0.10	0.00	0.000	<0.01 **
	Female	0.48	0.695		0.27	0.495	
ITEM4	Male	0.09	0.354	<0.05 *	0.10	0.316	0.34
	Female	0.23	0.460		0.00	0.000	
ITEM5	Male	0.40	0.570	<0.01 **	0.20	0.422	0.88
	Female	0.68	0.671		0.18	0.387	
ITEM6	Male	0.38	0.547	<0.05 *	0.00	0.000	<0.05 *
	Female	0.57	0.750		0.11	0.318	
ITEM7	Male	0.33	0.622	0.90	0.00	0.000	<0.01 **
	Female	0.34	0.569		0.20	0.457	
TOT	Male	2.07	2.097	<0.01 **	0.80	1.135	0.38
	Female	3.28	2.716		1.18	1.527	

* *p* < 0.05; ** *p* < 0.01.

## Data Availability

The data presented in this study are available on request from the corresponding author.
